# How are emotional distress and reassurance expressed in medical consultations for people with long-term conditions who were unable to receive curative treatment? A pilot observational study with huntington’s disease and prostate cancer

**DOI:** 10.1186/s40814-021-00833-z

**Published:** 2021-06-03

**Authors:** Niall C. Anderson, Yuefang Zhou, Gerry Humphris

**Affiliations:** School of Medicine, University of St Andrews, St. Andrews, KY16 9TF UK

**Keywords:** Affective reassurance, Cognitive reassurance, Emotional distress, Huntington’s disease, Prostate cancer

## Abstract

**Objective:**

It is unclear whether how people with long-term conditions express distress, and how clinicians respond, influences perceptions of consultation outcomes. The pilot study examined emotional distress and reassurance in consultations with people whose long-term conditions (at the time of consultations) were treated using active surveillance or symptom management (as no curative treatment was suitable).

**Methods:**

An observational pilot study was conducted involving consultations between people with long-term conditions and their respective clinician. Consultations between three clinicians (two Huntington’s Disease; one Prostate Cancer) and 22 people with long-term conditions (11 Huntington’s Disease; 11 Prostate Cancer) were audio-recorded. Participants also completed an expanded Consultation and Relational Empathy (CARE) Measure. Two researchers coded sessions using Verona Coding Definitions of Emotional Sequences (VR-CoDES/VR-CoDES-P). Code frequencies were calculated, *t* tests performed between conditions, and Pearson’s correlations performed for associations between CARE responses and clinician utterances.

**Results:**

People with long-term conditions expressed emotional distress on average 4.45 times per session, averaging 1.09 Concern and 3.36 Cue utterances. Clinicians responded with more explicit (2.59) and space-providing (3.36), than non-explicit (1.86) and space-reducing (1.09), responses per session. Clinicians expressed spontaneous reassurance on average 5.18 times per session, averaging 3.77 Cognitive and 1.5 Affective reassurance utterances. Huntington’s Disease consultations featured significantly more 'Cues', 'Concerns' and 'Overall' 'Emotional Distress', and 'Cognitive' and 'Overall' ‘Reassurance'.

**Conclusion:**

Emotional distress was expressed more using hints than explicit concern utterances. Clinicians predominantly explicitly explored distress rather than providing information/advice and provided advice using spontaneous cognitive reassurance. People with Huntington’s Disease expressed more concerns and received more reassurance, indicating different needs between conditions. Future research is required to explore emotional distress and reassurance in a larger sample of participants and long-term condition types, and how the practical implications of these findings may be used to enhance outcomes of consultations.

**Trial registration:**

N/A.

**Supplementary Information:**

The online version contains supplementary material available at 10.1186/s40814-021-00833-z.

## Key messages regarding feasibility

- What uncertainties existed regarding feasibility?

First, in what frequency and form is emotional distress expressed by people with long-term conditions, and how do clinicians respond and provide reassurance, in medical consultations for which curative treatment (at the time of the consultation) was not available? Second, how does this relate to people with long-term conditions’ perceptions of consultation outcomes, particularly in terms of consultation and relational empathy, and perceived satisfaction? Third, are differences present between different types of long-term health conditions for which curative treatments are not currently available?

- What are the key feasibility findings?

People with long-term conditions on average expressed emotional distress 4.45 times per session. Clinicians responded with more explicit (2.59) and space-providing (3.36), than non-explicit (1.86) and space-reducing (1.09), utterances. Clinicians expressed spontaneous reassurance on average 5.18 times per session (3.77 Cognitive; 1.5 Affective). Huntington’s disease consultations featured significantly more 'Cues', 'Concerns' and 'Overall' Emotional Distress, and 'Cognitive' and 'Overall' ‘Reassurance'. Consultations with a higher frequency of cognitive reassurance were associated with lower ratings of 'Letting you tell your story', ‘Showing care and compassion', 'Helping you to take control', and 'Making a plan of action with you'.

- What are the implications of the feasibility findings for the design of the main pilot study?

People with long-term conditions for which no curative treatment was available (at the time of the pilot study) demonstrated high levels of emotional distress in medical consultations, highlighting the need for further focus on this area within both healthcare research and intervention. The methodology used allowed for in-depth assessment of consultation interactions and provides valuable findings with real-world implications for healthcare policy, practice and training. Clinicians and people with long-term conditions for whom no curative treatment is currently available were highly interested and engaged in the research process. VR-CoDES and the CARE measure provided a suitable method for examining emotional distress, reassurance and how these may relate to consultation outcomes for consultations involving people with long-term conditions, and may allow direct comparisons to be made between conditions. However, developments in research relating to the investigation of sequencing of events since the pilot study was conducted would be beneficial for incorporating into future studies.

## Background

### Reassurance

A significant part of a clinician’s role in consultations involves managing concerns and providing reassurance about emotional distress that may be experienced about diagnosis, symptoms, treatment, and potential outcomes [1]. Reassurance is characterised as any clinician behaviour conducted to positively impact behaviour, thoughts or understanding [2]. Emotional distress and responses to reassurance may vary significantly based on age, perceived symptom discomfort and what this means to the person, clinicians’ working experience, and the timing (rather than frequency or duration) of reassurance [3–7]. Critically, the conversational style used by clinicians may promote different responses and outcomes. Open-ended questions and space-providing responses are associated with an Affective style that promotes relationship formation and exploration of both unmet needs and socio-emotional concerns [8]. This is crucial for building rapport and is associated with improvements in treatment adherence, self-efficacy, and condition-related distress. However, space-providing responses may warn of upcoming unpleasant events, highlight unmet needs, and is associated with reduced treatment satisfaction [9–12]. Because of aforementioned factors and consultations rarely providing exclusively Affective or Cognitive reassurance, reassurance is complex [8–12]. It requires a balance of providing sufficient space to voice concerns, while also asking closed-ended questions and providing space-reducing responses to sufficiently provide information and facilitate a change in beliefs [13].

Reducing emotional distress is an increasing focus of healthcare [14]. However, a limited evidence base is present regarding the reassurance needs of people with long-term conditions (LTC) in medical consultations [15]. While reassurance is assumed to associate with improved outcomes, parental expressions of empathy and reassurance during children’s dental procedures were associated with increased distress and poorer procedural outcomes [7, 16–18]. While this may potentially indicate reassurance triggered negative emotions through warning of unpleasant events [19], it may also potentially relate to which of two mutually exclusive types of reassurance was provided [20]. Affective reassurance involves creating rapport and showing empathy to reduce anxiety and enhance a sense of feeling understood. However, it may not empower people to develop new coping mechanisms and is associated with mixed outcomes. For example, improved satisfaction but negative effects on disease-related burden, symptom improvement, and adherence [1]. Conversely, Cognitive reassurance involves educating and providing knowledge to systematically impact beliefs. This approach may take longer and not have immediate positive effects, but is consistently associated with improved satisfaction, enablement and symptoms [1]. Hence, consultations favouring Affective reassurance are likely to be viewed as more empathic but Cognitive have more positive outcomes.

### Long-Term Conditions (LTC)

To understand the impact of reassurance on emotional distress, it is crucial to evaluate whether and how clinicians both respond to emotional distress and spontaneously provide reassurance [21]. Previous studies have assessed reassurance for emotional distress in settings such as head and neck cancer [12] and oncology [22]. However, little is currently known about emotional distress and reassurance in people whose LTC at the time of consultations are not actively curable and instead are treated using active surveillance and/or symptom management.

The pilot and feasibility study examined whether similarities and differences are present between two LTC: Huntington’s disease (HD) and prostate cancer (PC). These LTC were selected as being potentially suitable due to two primary factors. First, while there are significant differences between LTC in terms of their cause, presentation and treatment, for both people with HD (at all condition stages) and PC (for specific individuals at specific condition stages) curative treatment may not be available and consequently treatment may take the form of active surveillance and/or symptom management. Second, in the geographic area where the pilot study was conducted, strong links were present between the academic institution and the required relevant healthcare organisations, increasing practicality and feasibility.

HD is a progressive, monogenic, neurodegenerative disorder for which there is a 50% chance of passing HD on to ones’ children. Symptoms typically present from 25 to 44 years-of-age and average survival is 15–20 years post-onset [23–25]. HD progresses through five continuous stages, increasingly affecting behavioural, psychiatric, communicational, cognitive and sexual functioning [26, 27]. HD (currently) has no cure, and treatment involves managing symptoms through pharmaceutical, psycho-educational and/or neuropsychiatric treatments [25, 28–30]. Critically, as suicide prevalence may be as high as 13%—seven-to-twelve times greater than the general population [31]—understanding and managing emotional distress for diagnosis, symptoms and disease progression is crucial for HD consultations.

PC is characterised by prostate cells growing in an uncontrolled way and may result in prostate enlargement and urethra blockages [32]. PC is typically late-life onset and may take decades to progress through four stages from microscopic and undetectable, to affecting bones and tissue. At specific stages of PC, radiotherapy and/or surgery may be viable and necessary options. However, active surveillance is currently the prescribed treatment until such a time that symptoms and presentation indicate this may occur [33]. During active surveillance, people with PC may experience increased physiological and psychological difficulties associated with unmet needs including social support, coping and health-related quality-of-life [34]. Furthermore, while self-management is associated with improved functioning and emotional wellbeing, self-management requires the development of psychosocial strategies to reduce psychological, sexual and relationship problems post-diagnosis [35–37]. Hence, understanding and managing emotional distress during active surveillance is critical [38].

## Methods

### Pilot objectives

It is unclear whether how people with long-term conditions who are not currently suitable for curative treatment express distress, and how clinicians respond, influences perceptions of medical consultation outcomes. The pilot and feasibility study conducted in 2015 aimed to explore consultations between clinicians and people with LTC (specifically HD and PC) for which no active treatment was (currently) available, and instead, people with LTC were under active surveillance or symptom management. Specific objectives were to identify whether and how (1) people with LTC for whom curative treatment is not currently the indicated approach expressed emotional distress, (2) clinicians’ responded, (3) clinicians’ spontaneously expressed reassurance, (4) clinicians’ utterances related to perceptions of empathy and consultation satisfaction, and (5) differences were present between conditions.

A pilot and feasibility study was required and conducted due to a number of factors. First, while increased emphasis is being placed upon the importance of emotional distress and reassurance in medical consultations, there is currently a limited evidence base from which larger-scale studies may develop upon. Second, aforementioned studies typically focussed on individual LTC, and little is known about the similarities and differences in emotional distress and reassurance between different LTC, particularly those for which active surveillance or symptom management are the indicated treatment approach. Third, recruiting across different LTC populations and services can be challenging and an understanding is required of whether coding of sessions may receive buy-in from people with LTC and clinicians. Finally, while coding of emotional distress and reassurance has well defined methods, trialling these methods in different LTC particularly those for whom speech and/or physical disturbances may be present is important. Therefore, conducting a pilot and feasibility, rather than a larger-scale initial trial was deemed a suitable, feasible and practical approach to guide future research and practice.

### Participants

People with LTC and clinicians within a UK-based regional national healthcare authority’s HD or PC services were eligible. All participants were required to be aged ≥16 years old (legal age of capacity in Scotland (UK)), speak English, have no severe mental difficulties, and be physically and mentally able to consent. In order to balance potential HD communication difficulties with coding system requirements, provided people could verbally communicate (regardless of speech disturbances), they were eligible to participate. People with LTC were additionally required to be undergoing active surveillance or symptom management (not curative treatment). No further exclusions applied.

One PC (100% male; 38 years old) and two HD clinicians (100% female; 50–51 years old), and 22 people with LTC participated. Eleven people with HD encompassed 45% males, an average age of 41 years old (SD = 12), 45% undergoing active surveillance and 55% symptom management, and 82% completed the Consultation and Relational Empathy (CARE) measure [39, 40]. Eleven people with PC encompassed 100% males, an average age of 69 years old (SD = 6), 100% undergoing active surveillance, and 82% completed the CARE measure. Twenty-three consultations were audio or audio-visual recorded, with one lost due to a technical malfunction. Consultation average duration was 12 m41s (SD = 3m49s), with PC averaging 7m30s (SD = 3m16s) and HD 17m52s (SD = 1m8s).

### Procedure

An observational pilot and feasibility study was conducted involving consultations between people with LTC and their respective clinician. The pilot study involved audio-visual recordings (with audio recordings as back-ups) of standard medical consultations. Recordings were then coded for people with LTCs’ emotional distress expressions and clinicians’ responses. Effort was made to avoid influencing consultations context, structure or content. Clinicians were contacted, provided with information and invited to participate. Following informed verbal consent, clinicians identified suitable people with LTC to approach with information and an invitation to participate. Prior to recording consultations, the Principal Investigator (NA) provided information sheets and obtained informed written consent from all participants. The CARE measure [39, 40], with an additional consultation satisfaction question, was provided for voluntary completion and return using a pre-stamped envelope. Recordings were securely transferred, stored, transcribed, coded (NA), inter-rated coded (NA, YZ) and analysed in a locked, password-protected coding room and computer.

### Measures

Observer XT 10.5® [41] was used to code 17 (11 HD, 6 PC), and five audio-only PC recordings (due to a camera malfunction) were coded manually. Coding involved three stages. First, people with LTCs’ emotional distress verbal expressions were coded using validated Verona Coding Definitions of Emotional Sequencing (VR-CoDES) [42] (Table 1). As HD symptoms may include abnormal motor movements, to ensure consistency ‘*Cue F*’ was only coded for both conditions if crying/sobbing was present. Second, clinicians’ responses to cues/concerns were coded using validated VR-CoDES-of Provider Responses (VR-CoDES-P) [43] (Table 2). Third, spontaneous clinician presentations of Cognitive and Affective reassurance (in the absence of cues/concerns) were coded (Table 2) [1, 2, 20]. Coding reliability was tested, indicating ‘moderate’ intra-coder reliability (NA) (range: 79–81%; κ = 0.75–0.76) and 'moderate'-to-'strong' inter-coder reliability (NA; YZ) (range: 81–96%; κ = 0.77–0.95) [44]. The CARE measure [39, 40] was provided to generate a measure of people with LTCs’ ratings of empathy demonstrated by their clinician during the consultation. Questions on the 10-question CARE measure were scored on a five-point Likert scale ranging from '1 - Poor' to '5 - Excellent'. This provided individual scores for each question, and an overall perceived empathy score of 10–50. A further question, which asked 'Overall, how would you rate the consultation?’ was also added using the same Likert scale to assess overall consultation satisfaction.

**Table 1:** Definitions and examples of patients’ expressions of emotional distress

Code	Definitions	Examples
**Concern** *'A clear and unambiguous expression of an unpleasant emotion which was explicitly verbalized.'*	Emotional issue is recent or current	"It was a huge worry for me."
	Importance of the issue may or may not be stated	"I was concerned by that."
**Cue** *'A verbal or non-verbal hint which suggests underlying unpleasant emotions require clarification."*	(A) Vague or unspecified words or phrases used to describe one's emotions which would require clarification	"I just try to cope with it every day."
	(B) Non-explicit verbal hints towards one's hidden concerns	"I hope it is only going to be temporary."
	(C) Verbal expressions emphasizing negative emotional states regarding cognitive or physiological states	"I'll lie there and I'll have the alarm going off an hour before I get up."
	(D) Expressions referring to negative life events or conditions using neutral content which emphasizes emotional importance of issues and standards out from the narrative background	"I would say it's difficult, sometimes I just don't know what I'm doing half the time."
	(E) Repeated expressions of previously neutral emotions	“It has changed in the last fortnight, but then maybe, because of the medication…”
	(F) Non-verbal expressions of negative or unpleasant emotions	"I don't know... [Sobs]... it's hard."
	(G) Clear and unambiguous negative emotional expression in reference to the past (more than a month) or an unspecified time-frame	"Being down, I've never been down, you know, depressed, in my life like that."

**Table 2:** Clinician utterance categories and definitions

Utterance category	Code	Definitions	Examples	
**Response to emotional distress**	**Non-explicit** *'Affect or content of the cue/concern is not contained within the response'*	**Space-reducing** *'Response concerned with closing down further expansion for patient on cue/concern'*	Ignore	
			Shutting down	
			Information-advise	
		**Space-providing** *'Response concerned with eliciting further expansion from the patient on cue/concern'*	Silence	
			Backchannel	
			Acknowledge	
			Active invitation	
			Implicit empathy	
	**Explicit** *'Affect or content of the cue/concern is contained within the response"*	**Space-reducing** *'Response concerned with closing down further expansion for patient on cue/concern'*	Information-advise	
			Active blocking	
		**Space-providing** *'Response concerned with eliciting further expansion from the patient on cue/concern'*	Content	Acknowledge
				Explore
			Affect	Acknowledge
				Explore
				Empathy
**Spontaneous reassurance**	**Affective reassurance** *'Empathy'*	Spontaneous presentations (in the absence of patient cues/concerns) which aim to create rapport and promote an empathetic relationship	"I know, and you would have been such a good mum too."	
	**Cognitive reassurance** *'Information-advise'*	Spontaneous presentations (in the absence of patient cues/concerns) which aim to provide information and educate patients in order to achieve changes in beliefs or understanding	"If the side effects are too bothersome in the morning, if you feel, you know, quite parched, quite dry with the medication, stop it, it'll be fine."	

### Data analysis

Data was collated using Microsoft Office 2012 Excel™. Analyses were conducted using SPSS Statistical Packages 22™. Response frequency differences between LTC were assessed using *t* tests. For tests where the assumption of homogeneity of variance was violated, Welch-Satterthwaite adjustments were conducted. Pearson’s correlations were calculated between all forms of clinician responses and reassurance, and people with LTCs’ individual CARE measures [39, 40], overall CARE score and satisfaction.

## Results

### Emotional distress and clinicians’ responses

On average (Table 3), people with LTC expressed 3.36 cues and 1.09 concerns per session, and clinicians responded with 3.36 space-providing responses and 1.09 space-reducing responses utterances per session. Clinicians spontaneously provided 3.77 Cognitive and 1.5 Affective reassurance utterances per session. HD consultations featured significantly more emotional distress elicited by the person with LTC and their clinician, individual cues A & D, overall cues, concerns and non-explicit, explicit and space-providing responses to emotional distress. However, HD consultations featured significantly less cognitive and overall reassurance responses.

**Table 3:** Patients’ and clinicians’ utterance frequencies (*n* = 22 consultations)

Construct		Sample				Estimate of comparison (95%)
Code	Sub-domain	Huntington’s disease^**a**^	Prostate cancer^**a**^	Total	Mean per session	
**Patient emotional distress utterances**	**Cue A**	11 (85%)	2 (15%)	13	**0.59**	**.33 (.14, 1.50)**
	**Cue B**	14 (64%)	8 (36%)	22	1.00	.45 (**−**.42, 1.51)
	**Cue C**	6 (43%)	8 (57%)	14	0.64	.41 (**−**1.05, .68)
	**Cue D**	5 (100%)	0 (100%)	5	**0.23**	**.16 (.10, .81)**
	**Cue E**	0 (N/A)	0 (N/A)	0	0.00	N/A
	**Cue F**	1 (100%)	0 (0%)	1	0.05	.09 (**−**.11, .29)
	**Cue G**	15 (79%)	4 (21%)	19	0.86	.50 (**−**.07, 2.07)
	**Cue total**	52 (70%)	22 (30%)	74	**3.36**	**.86 (.88, 4.57)**
	**Concern**	19 (79%)	5 (21%)	24	**1.09**	**.55 (.11, 2.44)**
	**Patient-elicited**	46 (69%)	21 (31%)	67	**3.05**	**1.01 (.10, 4.44)**
	**Clinician-elicited**	25 (81%)	6 (19%)	31	**1.41**	**.48 (.71, 2.74)**
	**Cues/concerns total**	71 (72%)	27 (28%)	98	**4.45**	**1.08 (1.74, 6.26)**
**Clinician emotional distress response utterances**	**Non-explicit**	31 (76%)	10 (24%)	41	**1.86**	**.65 (.51, 3.31)**
	**Explicit**	40 (70%)	17 (30%)	57	**2.59**	**.86 (.30, 3.88)**
	**Space-providing**	54 (73%)	20 (27%)	74	**3.36**	**.92 (1.18, 5.00)**
	**Space-reducing**	17 (71%)	7 (29%)	24	1.09	.48 (**−**.09, 1.91)
	**Responses total**	71 (72%)	27 (28%)	98	**4.45**	**1.08 (1.74, 6.26)**
**Clinician spontaneous reassurance utterances**	**Affective reassurance**	11 (35%)	20 (65%)	31	1.5	.48 (**−**1.83, .19)
	**Cognitive reassurance**	32 (39%)	51 (61%)	83	**3.77**	**.71 (−3.22, −.23)**
	**Reassurance total**	43 (38%)	71 (62%)	114	**5.18**	**.82 (−4.25, −.84)**

^a^Frequency: raw number (percent total frequency)

### Relationship between clinicians' responses and perceived empathy and satisfaction

Eighty-two percent of participants (nine HD, nine PC) completed the CARE measure [39, 40]. Participants rated their clinician as 'Excellent' for 58%, ‘Very Good’ for 41%, and 'Good' for 1% of the 11 measures of perceived empathy and satisfaction. Significant negative correlations were present between Cognitive reassurance and the individual CARE measures of '*Letting you tell your story' (Estimate of comparison (95%) = .18 [[−.79, [−.1])*, '*Showing care and compassion' (Estimate of comparison (95%) = .16 [−.83, [−.24)]*, '*Helping you to take control' (Estimate of comparison (95%) = .19 [[−.83, [−.08])*, and *'Making a plan of action with you' (Estimate of comparison (95%) = .17 [[−.78, [−.1])*. Non-significant correlations were present between all individual CARE measures, the overall CARE score, the overall satisfaction score and all other measures.

## Discussion

### People with LTC emotional distress

Overall, people with LTC expressed 4.5 distress utterances per consultations. However, how emotional distress was expressed varied considerable. 'Concern' utterances are defined as 'Clear and unambiguous expressions of unpleasant emotions, whereas 'Cues' are defined as 'Verbal or non-verbal hints suggesting underlying unpleasant emotions which require clarification'. When all forms of cues were combined, people with LTC expressed over three-times as many cues (3.36) as concerns (1.09) per consultation. However, people with LTC expressed more clear and unambiguous (concern) expressions of unpleasant emotions than any individual cue. Furthermore, when people with LTC did express a cue, this was most frequently through 'Non-explicit hints towards hidden concerns' (1.00), 'Negative emotional expressions in reference to the past' (0.86), 'Verbal expressions of negative emotions regarding physical or cognitive states' (0.64) or 'Vague or unspecified utterances regarding ones' emotions' (0.59). People with LTC rarely expressed emotional distress through 'Negative life events through neutral content' (0.23) or 'Non-verbal expressions of negative emotions' (0.05), and never through 'Repeated expression of previously neutral emotions' (0.00).

The type and frequency of cues and concerns have implications for understanding emotional distress in these LTC populations. First, while consultations were primarily conducted for health and medical needs, the high levels of emotional distress supports the importance of also accounting for emotional and psychological concerns during consultations [45]. Additionally, as emotional distress was twice as likely to be elicited by people with LTC than clinicians, having the knowledge and experience to reactively support and enable people with LTC to cope with emotional distress should form a key part of consultations and consequently clinician training [14]. Second, approximately one concern being expressed per session indicates people with LTC were able to use sessions as an opportunity to express distress. However, as over three times more cues were present people with LTC were more likely to hint at concerns rather than directly voicing them. From people with LTC perspective, this may reflect experiencing emotional distress but feeling unclear whether they can, should and/or are able to reflect this in sessions. From clinicians’ perspective, as cues are hints, this may potentially make distress more difficult to detect and respond to, particularly if this is not the focus of consultations [46]. This may be supported by the most frequent clinician responses being explicit and space-providing responses, which may indicate a need to evoke further information to seek clarification. Third, when people with LTC expressed cues they rarely used non-verbal expressions of negative emotions (one participant cried), and never did so through repeatedly expressing previously neutral content. Therefore, despite people with LTC preferring to hint at concerns rather than directly expressing them, they used a format that is verbally non-neutral which may potentially increase the salience of the cue and invoke a response from clinicians.

While distress was high across participants, several factors may have influenced why people with HD expressed over twice as many overall, patient-elicited, and clinician-elicited distress utterances than people with PC per session. First, as HD consultations were on average over twice as long (17m52s) as PC consultations (7m30s), the additional time may have allowed greater opportunity to express distress, explore topics further and/or create rapport with clinicians. However, this possibility is reduced by both samples demonstrating emotional distress more frequently towards the beginning of consultations. Second, in addition to overall emotional distress, people with HD expressed significantly more concerns, overall cues, 'Vague or unspecified utterances regarding ones' emotions' and 'Negative life events through neutral content' utterances. While both conditions are associated with significant challenges for quality-of-life, the increased focus on negative life events may relate to the progressive, neuro-degenerative nature of HD. In particular, this may potentially indicate disinhibited behaviour relating to neurological and personality changes or potential implications for genetic transmission [23]. Third, emotional distress differences may potentially relate to differences in consultation focus, content or clinician style, such as clinicians' responses to emotional distress and spontaneous use of empathy. Therefore, future research is required to understand the mechanisms behind the high levels of emotional distress in people with LTC, distress typically being expressed using hints rather than directly voicing concerns and between LTC differences.

### Clinicians’ reassurance

Overall, clinicians expressed 5.18 spontaneous reassurance utterances per consultation. While consultations were primarily conducted for medical difficulties, this also demonstrates clinicians used them as critical opportunities to reassure people with LTC about emotional distress rather than focusing purely on medical issues. Previous research [47] demonstrated in breast cancer consultations clinicians undergo different cognitive states during which they are more likely to use a specific type of reassurance and less likely to switch types. This also appeared to be reflected in this pilot study as spontaneous reassurance was more than twice as likely to be expressed through Cognitive (3.77) utterances which 'aim to provide information and educate patients in order to achieve changes in beliefs or understanding', than it was to be through Affective (1.5) utterances which 'aim to create rapport and promote and empathetic relationship'. The preference for Cognitive reassurance to systematically provide information contrasted with how clinicians responded to emotional distress, which was more frequently through Explicit (2.59) and/or space-providing responses (3.36) to seek expansion upon affect relating to distress, than Non-Explicit (1.86) and/or space-reducing responses (1.09) to provide information and change beliefs.

Future research is required to explore the potential factors that may have influenced why consultations demonstrated greater levels of Cognitive than Affective reassurance, and spontaneous reassurance than responses to emotional distress. First, as more space-providing than space-reducing responses were present, clinicians may potentially seek to explore distress further to increase their understanding of difficulties, before then providing reassurance if/when deemed necessary. In other words, this may potentially be a response to people with LTC expressing more hints (cues) than concerns, resulting in clinicians seeking expansion before deciding how to respond. Second, reassurance may be used to pre-emptively managing and helping people with LTC to cope with distress before they feel the need to express it. As reassurance most frequently occurred towards the beginning of consultations in Cognitive form, this may mean that clinicians seek to provide spontaneous reassurance early as a pre-emptive attempt to reduce distress. Third, clinicians may frequently not perceive emotional distress utterances as being salient and so await content indicating reassurance is required [48].

Clinicians of both LTC expressed more Cognitive than Affective reassurance. However, HD clinicians expressed nearly half as many Affective and two thirds as many Cognitive reassurance utterances as PC clinicians. Future research is required to develop upon pilot findings and examine whether this relates to aforementioned between-LTC differences influencing needs and consequently clinician responses, or whether further possible explanations are present, as this may have implications for practice.

As all people with PC were undergoing active surveillance, clinicians may potentially have provided high levels of Affective reassurance to create rapport and Cognitive reassurance to provide disease-related information in previous appointments. Conversely, people with HD were at different condition and service involvement stages, the prevalence of HD in the general population is lower than PC, and consequently HD clinicians may have had greater capacity/remit to have increased involvement (indicated by greater session durations). This may have been associated with increased opportunity to develop rapport and consequently HD clinicians perceiving increased self-efficacy to focus more on Cognitive reassurance and/or tailoring their reassurance approach to individuals’ needs. Furthermore, as people with HD demonstrated more emotional distress utterances than people with PC, this may have provided greater opportunity to respond to the issue directly and reduced reassurance needs. Whereas for PC consultations where the frequency of emotional distress was limited, spontaneous reassurance may have been used to compensate. Therefore, future research should seek to examine the links between emotional distress and spontaneous reassurance, including whether factors associated with specific LTC and services influences needs and responses.

Based on previous research, it would be expected that consultations demonstrating higher levels of Cognitive reassurance would result in higher levels of satisfaction [13], while those with higher levels of Affective reassurance would result in higher levels of perceived empathy [11]. Overall, no correlation was present between Affective or Cognitive reassurance and satisfaction. However, while no correlations were present between Affective reassurance and any empathy measure, higher Cognitive reassurance was significantly associated with lower scores for 'Letting you tell your story' , 'Showing care and compassion' , 'Helping you to take control' and 'Making a plan of action with you'. This could potentially indicate that, while Cognitive reassurance serves the purpose of providing information with a view to reduce distress and change beliefs, providing more utterances may be perceived as less empathetic. Conversely, due to LTC symptoms, diagnosis and symptom management having significant physical, psychological and social burden, it may potentially be that Cognitive reassurance involves the provision of necessary but challenging information to process and consequently is not perceived as empathetic. However, as 99.49% of measures were scored as 'Very Good' or 'Excellent', reassurance formed a crucial part of consultations and future research would benefit from exploring the optimal reassurance approach.

### Pilot study strengths and limitations

To the researchers' knowledge, the pilot and feasibility study was the first to explore clinician management of emotional distress specifically in people with LTC where no treatment was available and instead were undergoing active surveillance or symptom management. The pilot study benefitted from all recruited people with LTC completing their medical consultation and 82% returning their CARE measure. Additionally, all clinicians voluntarily agreed to participate and actively engaged in recruiting participants. This indicates that both clinicians and people of these LTC populations and services, for whom active surveillance or symptom management was the only (current) option available, were highly interested and engaged in research with potential implications for care. Additionally as moderate intra-rater and moderate-to-strong inter-rater reliability was present, the people with LTC and clinician codes and results can be stated with a reasonable degree of certainty. Furthermore, rather than merely assessing whether emotional distress or reassurance was present in consultations, the pilot study also assessed the form that distress was expressed in how clinicians responded and how this relates to spontaneous reassurance. Finally, the pilot study assessed how spontaneous reassurance related to people with LTCs’ perceptions of clinician empathy and consultation satisfaction. Therefore, the methodology used allowed for in-depth assessment of consultation interactions and provides valuable findings with real-world implications for healthcare policy, practice and training.

The pilot and feasibility study featured two study sites, two LTC populations, three clinician and 22 people with LTC participants. This was deemed practical for a pilot and feasibility study, and the populations and settings targeted were both accessible and viewed as priorities for the UK-based regional national healthcare authority. However, a number of limitations (which are common findings of pilot and feasibility studies) were present which must be accounted for and developed upon in future, larger-scale studies. First, while clinicians offering participation to people with LTC within their clinics was deemed an appropriate and suitable approach for facilitating participation by a trusted and knowledgeable source, this raises the possibility of a recruitment bias. Second, in terms of the participants recruited, multiple confounding factors may have influenced outcomes including the gender and average age of people with LTC (HD: 55% female; 41 years old; PC: 0% female, 69 years old) and clinicians (HD: 100% female, 50–51 years old; PC: 0% female, 38 years old), current treatment (HD: 45% active surveillance; PC 100% active surveillance) and average session duration (HD: 17m52s; PC: 7m30s). Additionally, a number of statistical tests were conducted with a limited sample size. Therefore, future studies would benefit from an increased sample size of people with LTC and clinicians, and the inclusion of a control condition, such as a previously explored area of reassurance research or check-up appointments which reveal no diagnosis, in order to increase the reliability of findings and make valid comparisons.

Several implications are present from the pilot and feasibility study utilizing the validated VR-CoDES, VR-CoDES-P and CARE measures [38, 40–43]. First, while the CARE measure is widely used in healthcare research, the addition of a satisfaction question may not have been sufficient to detect effects and future research would benefit from the development of a more expansive questionnaire. Second, the Observer XT® system provided a systematic method for assessing audio-visual observations. However, unfortunately visual data was lost for five of 22 recordings, and as the system did not allow for audio-only files, these were coded manually without visual data being detected.

The pilot study was conducted in 2014–2015 using VR-CoDES. VR-CoDES are used as a validated measure for assessing emotion distress across multiple domains [12, 18, 41, 42]. While the action-response criteria utilised was beneficial for exploring potential bi-direction relationships in people with LTC-clinician interactions, it did not originally account for the occurrence of potentially important information that may influence emotional distress or implications for subsequent codes. However, research has since explored how VR-CoDES may be used to conduct analysis of longer sequences of codes to negate this confound [47, 49, 50]. In the future, researchers may wish to consider the sequence of events when using a form of reassurance to understand the presentation of distress in the form of cues or concerns, and types of reassurance, in order to further understand and assist the development of theoretical models for testing [47]. Therefore, future replications are required which utilize pilot and feasibility study strengths, minimize limitations, and factor in subsequent research advancements.

## Conclusion

To the authors’ knowledge, the pilot study was the first to examine emotional distress and reassurance in medical consultations with people with LTC for whom active surveillance or symptom management was the indicated approach. The pilot study demonstrated actively engaging clinicians and people with LTC in healthcare-focussed research may enhance the understanding of both interactions and self-rated outcomes of sessions. The pilot study highlighted the importance of understanding emotional distress, clinicians’ responses and reassurance in consultations and may indicate that a ‘one-size-fits-all’ approach to research and LTC management may not account for nuanced differences between LTC. Future large-scale studies are required which develop upon pilot findings and recommendations, and incorporate subsequent research developments, with larger participant samples and variability.

## Supplementary Information

**Additional file 1.**

## Data Availability

The datasets used and/or analysed during the pilot study are available as a supplementary file.

## Abbreviations

LTC: Long-term conditions
HD: Huntington’s disease
PC: Prostate cancer
CARE: Consultation and Relational Empathy
VR-CoDES: Verona Coding Definitions of Emotional Sequences
VR-CoDES-P: Verona Coding Definitions of Emotional Sequences of Provider Responses

## References

[1] Pincus T, Holt N, Vogel S, Underwood M, Savage R, Walsh DA, Taylor SJC (2013). Cognitive and affective reassurance and patient outcomes in primary care: a systematic review. Pain.

[2] Linton SJ, McCracken LM, Vlaeyen JW (2008). Reassurance: help or hinder in the treatment of pain. Pain.

[3] Gonzalez JC, Routh DK, Armstrong FD (1993). Effects of maternal distraction versus reassurance on children's reactions to injections. J Pediatr Psychol.

[4] Jay SM, Ozolins M, Elliott CH, Caldwell S (1983). Assessment of children’s distress during painful medical procedures. Health Psychol.

[5] Piira T, Taplin JE, Goodenough B, Von Baeyer CL (2002). Cognitive-behavioural predictors of children’s tolerance of laboratory-induced pain: implications for clinical assessment and future directions. Behav Res Ther.

[6] Warwick HM, Salkovskis PM (1985). Reassurance. Br Med J.

[7] Zhou Y, Cameron E, Forbes G, Humphris G (2011). Systematic review of the effect of dental staff behaviour on child dental patient anxiety and behaviour. Patient Educ Couns.

[8] Mikesell L (2013). Medicinal relationships: caring conversation. Med Educ.

[9] Kim SS, Kaplowitz S, Johnston MV (2004). The effects of physician empathy on patient satisfaction and compliance. Eval Health Prof.

[10] Norfolk T, Birdi K, Walsh D (2007). The role of empathy in establishing rapport in the consultation: a new model. Med Educ.

[11] Zachariae R, Pedersen CG, Jensen AB, Ehrnrooth E, Rossen PB, Von Der Maase H (2003). Association of perceived physician communication style with patient satisfaction, distress, cancer-related self-efficacy, and perceived control over the disease. Br J Cancer.

[12] Zhou Y, Humphris G, Ghazali N, Friderichs S, Grosset D, Rogers SN (2015). How head and neck consultants manage patients’ emotional distress during cancer follow-up consultations: a multilevel study. Eur Arch Otorhinolaryngol.

[13] Hesson AM, Sarinopoulos I, Frankel RM, Smith RC (2012). A linguistic study of patient-centered interviewing: emergent interactional effects. Patient Educ Couns.

[14] Absolom K, Holch P, Pini S, Hill K, Liu A, Sharpe M, Richardson A, Velikova G, on behalf of the NCRI COMPASS Supportive and Palliative Care Research Collaborative (2011). The detection and management of emotional distress in cancer patients: the views of health-care professionals. Psycho-oncology.

[15] Khan NF, Evans J, Rose PW (2011). A qualitative study of unmet needs and interactions with primary care among cancer survivors. Br J Cancer.

[16] Blount RL, Corbin SM, Sturges JW, Wolfe VV, Prater JM, James LD (1989). The relationship between adults’ behavior and child coping and distress during BMA/LP procedures: a sequential analysis. Behav Ther.

[17] Dahlquist GG, Ivarsson S, Lindberg B, Forsgren M (1995). Maternal enteroviral infection during pregnancy as a risk factor for childhood IDDM: a population-based case-control study. Diabetes.

[18] Zhou Y, Humphris GM (2014). Reassurance and distress behavior in preschool children undergoing dental preventive care procedures in a community setting: a multilevel observational study. Ann Behav Med.

[19] McMurtry CM, McGrath PJ, Chambers CT (2006). Reassurance can hurt: parental behavior and painful medical procedures. J Pediatr.

[20] Coia P, Morley S (1998). Medical reassurance and patients’ responses. J Psychosom Res.

[21] Pruyn JF, Heule-Dieleman HA, Knegt PP, Mosterd FR, Van Hest MA, Sinnige HA (2004). On the enhancement of efficiency in care for cancer patients in outpatient clinics: an instrument to accelerate psychosocial screening and referral. Patient Educ Couns.

[22] Ford S, Fallowfield LJ, Lewis S (1994). Can oncologists detect distress in their out-patients and how satisfied are they with their performance during bad news consultations?. Br J Cancer.

[23] Arrasate M, Finkbeiner S (2012). Protein aggregates in Huntington’s disease. Exp Neurol.

[24] Paulsen JS, Hoth KF, Nehl C, Stierman L, Huntington Study Group (2005). Critical periods of suicide risk in Huntington’s disease. Am J Psychiatry.

[25] Ross CA, Tabrizi SJ (2011). Huntington’s disease: from molecular pathogenesis to clinical treatment. Lancet Neurol.

[26] Vonsattel JPG, DiFiglia M (1998). Huntington disease. J Neuropathol Exp Neurol.

[27] Walker FO (2007). Huntington's Disease. Lancet.

[28] Craufurd D, Snowden J (2002). Neuropsychological and neuropsychiatric aspects of Huntington’s disease. Oxford Monog Med Genet.

[29] Hamilton JM, Salmon DP, Corey-Bloom J, Gamst A, Paulsen JS, Jerkins S, Jacobson MW, Peavy G (2003). Behavioural abnormalities contribute to functional decline in Huntington’s disease. J Neurol Neurosurg Psychiatry.

[30] Thompson JC, Harris J, Sollom AC, Stopford CL, Howard E, Snowden JS, Craufurd D (2012). Longitudinal evaluation of neuropsychiatric symptoms in Huntington’s disease. J Neuropsychiatr Clin Neurosci.

[31] Farrer LA, Opitz JM, Reynolds JF (1986). Suicide and attempted suicide in Huntington disease: implications for preclinical testing of persons at risk. Am J Med Genet.

[32] Berger MF, Lawrence MS, Demichelis F (2011). The genomic complexity of primary human prostate cancer. Nature.

[33] Giovannucci E, Liu Y, Platz EA, Stampfer MJ, Willett WC (2007). Risk factors for prostate cancer incidence and progression in the health professionals follow-up study. Int J Cancer.

[34] Paterson C, Jones M, Rattray J, Lauder W (2013). Exploring the relationship between coping, social support and health-related quality of life for prostate cancer survivors: a review of the literature. Eur J Oncol Nurs.

[35] Chien CH, Liu KL, Chien HT, Liu HE (2014). The effects of psychosocial strategies on anxiety and depression of patients diagnosed with prostate cancer: a systematic review. Int J Nurs Stud.

[36] Chisholm KE, McCabe MP, Wootten AC, Abbott JAM (2012). Psychosocial interventions addressing sexual or relationship functioning in men with prostate cancer. J Sex Med.

[37] Cockle-Hearne J, Faithfull S (2010). Self-management for men surviving prostate cancer: a review of behavioural and psychosocial interventions to understand what strategies can work, for whom and in what circumstances. Psycho-oncology.

[38] Dall’Era MA, Albertsen PC, Bangma C, Carroll PR, Carter HB, Cooperberg MR, Freedland SJ, Klotz LH, Parker C, Soloway MS (2012). Active surveillance for prostate cancer: a systematic review of the literature. Eur Urol.

[39] Bikker AP, Fitzpatrick B, Murphy D, Mercer SW (2015). Measuring empathetic, person-centred communication in primary care nurses: validity and reliability of the Consultation and Relational Empathy (CARE) Measure. BMC Fam Pract.

[40] Mercer SW, Maxwell M, Heaney D, Watt G (2004). The consultation and relational empathy (CARE) measure: development and preliminary validation and reliability of an empathy-based consultation process measure. Fam Pract.

[41] Zimmerman PH, Bolhuis JE, Willemsen A, Meyer ES, Noldus LP (2009). The Observer XT: a tool for the integration and synchronization of multimodal signals. Behav Res Methods.

[42] Zimmermann C, Del Piccolo L, Bensing J, Bergvik S, De Haes H, Eide H (2011). Coding patient emotional cues and concerns in medical consultations: the Verona coding definitions of emotional sequences (VR-CoDES). Patient Educ Couns.

[43] Del Piccolo L, De Haes H, Heaven C, Jansen J, Verheul W, Bensing J (2011). Development of the Verona coding definitions of emotional sequences to code health providers’ responses (VR-CoDES-P) to patient cues and concerns. Patient Educ Couns.

[44] Cohen J (1960). A coefficient of agreement for nominal scales. Educ Psychol Meas.

[45] Sisk BA, Friedrich AB, DuBois J, Mack JW (2020). Emotional communication in advanced pediatric cancer conversations. J Pain Symp Manage.

[46] Johnson Shen M, Ostroff JS, Hamann HA, Haque N, Banerjee SC, McFarland DC (2019). Structured analysis of empathic opportunities and physician responses during lung cancer patient-physician consultations. J Health Commun.

[47] Popov V, Ellis-Robinson A, Humphris G (2019). Modelling reassurances of clinicians with hidden Markov models. BMC Med Res Methodol.

[48] Van Vliet LM, Francke AL, Westendorp J, Hoffstädt H, Evers AW, Van der Wall E (2019). The use of expectancy and empathy when communicating with patients with advanced breast cancer; an observational study of clinician-patient consultations. Front Psychiat.

[49] Del Piccolo L, Finset A, Mellblom AV, Figueiredo-Braga M, Korsvold L, Zhou Y (2017). Verona coding definitions of emotional sequences (VR-CoDES): conceptual framework and future directions. Patient Educ Couns.

[50] Mellblom AV, Korsvold L, Ruud E, Lie HC, Loge JH, Finset A (2016). Sequences of talk about emotional concerns in follow-up consultations with adolescent childhood cancer survivors. Patient Educ Couns.

